# Vitamin B_12b_ Enhances the Cytotoxicity of Diethyldithiocarbamate in a Synergistic Manner, Inducing the Paraptosis-Like Death of Human Larynx Carcinoma Cells

**DOI:** 10.3390/biom10010069

**Published:** 2020-01-01

**Authors:** Marina Solovieva, Yuri Shatalin, Roman Fadeev, Olga Krestinina, Yulia Baburina, Alexey Kruglov, Ekaterina Kharechkina, Margarita Kobyakova, Vadim Rogachevsky, Elena Shishkova, Vladimir Akatov

**Affiliations:** 1 Institute of Theoretical and Experimental Biophysics, Russian Academy of Sciences, Pushchino, 142290 Moscow Region, Russia; m_solovieva@iteb.ru (M.S.); yury.shatalin@yandex.ru (Y.S.); fadeevrs@gmail.com (R.F.); ovkres@mail.ru (O.K.); byul@rambler.ru (Y.B.); krugalex@rambler.ru (A.K.); katya.kypri@gmail.com (E.K.); ritaaaaa49@gmail.com (M.K.); 2 Institute of Cell Biophysics, Russian Academy of Sciences, Pushchino, 142290 Moscow Region, Russia; vadim_rogachevsky@synapsis.ru (V.R.); shishkova@neuro.nnov.ru (E.S.)

**Keywords:** hydroxycobalamin, vitamin B_12b_, diethyldithiocarbamate, synergism, cytotoxicity, endoplasmic reticulum, ER-stress, cytoplasm vacuolization, paraptosis-like cell death

## Abstract

We have shown that hydroxycobalamin (vitamin B_12b_) increases the toxicity of diethyldithiocarbamate (DDC) to tumor cells by catalyzing the formation of disulfiram (DSF) oxi-derivatives. The purpose of this study was to elucidate the mechanism of tumor cell death induced by the combination DDC + B_12b_. It was found that cell death induced by DDC + B_12b_ differed from apoptosis, autophagy, and necrosis. During the initiation of cell death, numerous vacuoles formed from ER cisterns in the cytoplasm, and cell death was partially suppressed by the inhibitors of protein synthesis and folding, the IP3 receptor inhibitor as well as by thiols. At this time, a short-term rise in the expression of ER-stress markers BiP and PERK with a steady increase in the expression of CHOP were detected. After the vacuolization of the cytoplasm, functional disorders of mitochondria and an increase in the generation of superoxide anion in them occurred. Taken together, the results obtained indicate that DDC and B_12b_ used in combination exert a synergistic toxic effect on tumor cells by causing severe ER stress, extensive ER vacuolization, and inhibition of apoptosis, which ultimately leads to the induction of paraptosis-like cell death.

## 1. Introduction

It is known that vitamin B_12_ is necessary for the metabolism of humans; it is used in the treatment of neurological, psychiatric, and toxicological diseases, as well as in anemia. Its effects have been studied in other pathologies, such as refractory hypotension, vasoplegia, and optic neuropathy [1,2,3,4,5]. Vitamins of group B_12_ participate as cofactors in hematopoiesis, the regulation of the metabolism of other vitamins, nitrous bases, amino acids, and fatty acids and affect gene expression [6,7]. There is evidence indicating that rapidly proliferating tumor cells and bacteria have increased demands for vitamin B_12_ which is due to the fact that cobalamin is a cofactor of 5-methyltetrahydrofolate-homocysteine methyl transferase, a key enzyme of the synthesis of deoxyribonucleotides [8]. Based on this evidence, studies devoted to possible application of B_12_ in antitumor therapy are being carried out. Walker and coworkers showed that the deficiency of vitamin B_12_ leads to the apoptosis of tumor cells, and nonproliferating cells are not damaged in this case [9]. Bauer with coworkers found that the B_12_ derivative nitrosylcobalamin in combination with interferon has a pronounced toxic effect on tumor cells [10]. Studies are in progress in an attempt to design drugs of directed action based on conjugates of B_12_ with known antitumor drugs [11,12]. It has been shown earlier that cobalamins are capable of catalyzing the formation of reactive oxygen species in the presence of natural reducing agents, e.g., ascorbate and thiols, which leads to oxidative stress [13,14,15]. We have found that hydroxycobalamin (B_12b_) in combination with ascorbate and thiol antioxidants GSH, NAC, and DTT catalyzes the formation and accumulation of hydrogen peroxide in medium, inducing single- and double-strand DNA breaks and the damage to lysosomes, which ultimately led to apoptoptic cell death [16,17,18,19]. Recently, we have shown that B_12b_ catalyzes the oxidation of diethyldithiocarbamate (DDC), a member of another group of thiol-containing substances, namely dithiocarbamates (DTC) [20]. DTC are used in different spheres of human activity, in particular, as catalyzers in industry and as toxic chemicals in agriculture. DDC, which is a chelator of metal ions, is used for detoxication after nickel and cadmium poisoning. It is believed that the cytotoxicity of DDC is mainly associated with its ability to chelate the transition metal ions, primarily copper and zinc [21,22,23,24,25,26,27]. Based on this feature, novel complexes of DDC and other DTC with metal ions for the application in antitumor therapy are being designed [28,29,30]. We have revealed earlier the ability of B_12b_ combined with DDC to catalyze the formation of disulfiram (DSF) oxidized derivatives, which caused a significant increase in DDC cytotoxicity independently of copper ions [20]. The goal of the present study was to elucidate the mechanism underlying the increase in the cytotoxic effect of DDC in combination with B_12b_ on tumor cells.

## 2. Materials and Methods

### 2.1. Chemicals

DDC and 1,10-phenanthroline (PTL) were purchased from MPbiomedicals (Irvine, CA, USA); Hoechst 33342 (H342), LysoTracker^®^ Green DND-26, MitoTracker Green, MitoTracker Deep Red, and ER-Tracker Red were from Molecular Probes Inc. (Carlsbad, CA, USA); and fetal bovine serum was from Gibco (Carlsbad, CA, USA). Other chemicals were from Sigma (Milwaukee, WI, USA).

### 2.2. Cell Culture

Human epidermoid larynx carcinoma HEp-2, human lung carcinoma A549, human squamous carcinoma A431, human fibrosarcoma HT1080, human colon adenocarcinoma HT29 cell lines were obtained from the Russian Cell Culture Collection (Institute of Cytology, Russian Academy of Sciences, St. Petersburg, Russia). Cells were grown in DME medium supplemented with 10% FBS, 80 mg/l of gentamycin, and 20 mM sodium bicarbonate at 37 °C in an atmosphere of 5% CO_2_. All cell lines were confirmed to be free of mycoplasma infection through regular testing by Hoechst 33342 (H342) staining.

### 2.3. Cytotoxicity Assay and Drug Treatment

Cells were seeded in 96-well microplates or culture dishes (Corning, NY, USA) at a concentration of 2 × 10^5^ cells/mL (2 × 10^4^ cells in 100 μL/well). Freshly prepared solutions of the vitamin B_12b_ and filtered DDC were added 24 h after cell seeding. Chelators and inhibitors (cycloheximide (CHX), zVAD.fmk, 4-phenylbutyric acid (4-PBA), 2-Aminoethyl diphenylborinate (2-APB), EGTA, desferrioxamine (DFO), 1,10-phenanthroline (PTL)) were added to the cultures 90 min before the administration of DDC and B_12b_. 3-methyladenine (3-MA), Bafilomycin A1, and chloroquine (CQ) were added simultaneously with DDC + B_12b_. After 6-h incubation, the medium was replaced by a fresh growth medium without additives. Cytotoxicity was determined using the crystal violet cytotoxicity assay by the ratio of optical densities at 620 nm in treated and untreated cultures at 48 h after the addition of B_12b_ and DDC. Cell viability was estimated by the trypan blue exclusion assay after the trypsinization of cell culture. The combination index was calculated according to [31].

### 2.4. DNA Fragmentation Assay

For the detection of internucleosomal DNA cleavage, DNA samples of 5 × 10^5^ cells were subjected to electrophoresis in 1.2% agarose gel as described earlier [18]. Gels were stained for 20 min with ethidium bromide (0.5 μg/mL).

### 2.5. Assay of the Activity of Caspase-3

The activity of caspase-3 was assayed by the fluorescence of cells using the fluorogenic substrate Acetyl-Asp-Glu-Val-Asp-7-Amino-4-methylcoumarin (DEVD-AMC; Enzo Life Sciences, Farmingdale, NY, USA) according to manufacturer’s recommendations. TRAIL (TNF alpha Related Apoptosis Inducing Ligand)-treated cells were used as caspase 3 positive control. Fluorescence was estimated on a multiplate reader Infinite 200 (TECAN, Grödig, Austria).

### 2.6. Transmission Electron Microscopy

НЕр-2 cell cultures were fixed with a 2.5% glutaraldehyde solution in 0.1 M Na-cacodylate buffer for 1 h, washed twice with the same buffer (for 10 min each), and postfixed for 1 h in 1% OsO_4_ reduced with 1% (*w*/*w*) potassium ferricyanide (K_3_Fe[CN]_6_) in 0.1 M Na-cacodylate. Fixed cells were washed with distilled water, dehydrated in graded ethanol (40, 60, 80, 3 × 100%, for 10–15 min each), and submerged in mix 1:1 (*v*/*v*) of DER resin (DER-332/ DER-732/DDSA) with 100% ethanol for 30 min at room temperature. The resin/ethanol mixture was replaced with 100% resin and leaved in an oven at 37 °C for 30 min and then at 60 °C for 48 h. Short series of 70-nm sections were prepared from the middle part of the cell layer with a diamond knife on a Leica UC7 ultramicrotome, picked up on piloform and carbon coated single slot grids, stained with uranyl acetate and a modified triple Sato lead stain, and imaged on a JEM-1400 electron microscope in a large montage acquisition mode with the SerialEM software at magnification ×6K (res. 1.8 nm/pixel). Sets of 8 × 8 frames were elastically stitched into each montage of about 50 × 30 µm in size (covering several HEp-2 cells) with TrakEM2 (ImageJ, https://imagej.net) after which serial montages were elastically aligned and analyzed.

### 2.7. Fluorescent Confocal Microscopy

Cells were seeded on cover glasses inside a Petri dish as described earlier. Twenty-four hours after seeding, control cultures and cultures treated with DDC + B_12b_ were stained with fluorescent dyes according to manufacturers’ recommendations. Images were obtained using a TCS SP5 confocal microscope (Leica Microsystems, Mannheim, Germany) and analyzed by the Leica Application Suite Advanced Fluorescence 2.1.0 software (Leica Microsystems).

### 2.8. Flow Cytometry Assay

The formation of autophagosomes and autolysosomes was assessed using the acridine orange (AO) [32]. Cells were first treated with DDC and DDC+B_12b_ for 1–4 h and then washed and stained with an AO solution (5 μg/mL) in complete medium for 15 min. Changes in red (FL3) fluorescence were determined by a BD Accuri C6 flow cytometer (BD Biosciences, San Jose, CA, USA).

The mitochondrial potential was estimated by the cell-permeant green-fluorescent lipophilic dye 3,3’-dihexyloxacarbocyanine iodide DiOC_6_(3), which accumulates in mitochondria due to their large negative membrane potential. Briefly, after incubation, cultures were trypsinized, washed with warm DPBS, stained with DiOC_6_(3) (10 nM) for 15 min at 37 °C in the dark, and washed twice with prewarmed (37 °C) growth medium. The fluorescence of DiOC_6_(3) was measured on the FL1 channel. The disappearance of ΔΨ_m_ in the presence of 100 μM ClCCP served as a positive control. The generation of superoxide anion was assayed on the FL3 channel after the same procedure of preparation, staining with 1 µM MitoSOX, and washing of cells in PBS [33]. The intracellular oxidative activity and the concentration of intracellular calcium were evaluated using 2’,7’-dichlorodihydrofluorescein diacetate (DCHFDA, 20 μM) and Fluo-4 AM (5 μM) on the FL1 channel, and the loading cells with dyes is described above.

### 2.9. Ca^2+^-Retention Capacity of Mitochondria in Permeabilized Cells

Mitochondrial Ca^2+^ uptake and release was recorded in a temperature-controlled electrode chamber using a Ca^2+^-electrode connected to a computerized recording system Record 4 (Institute of Theoretical and Experimental Biophysics, RAS, Pushchino, Russia) [34]. Cells (10^6^/mL) were treated with digitonin at a concentration of 30 μg/mL for the permeabilization of the plasma membrane. The loading capacity was defined as the amount of Ca^2+^ mitochondria take up in small pulses before Ca^2+^ is released.

### 2.10. Measurement of Intracellular ATP

ATP in cells was measured using an ATP Biomass Kit HS (BioThema AB, Stockholm, Sweden) in accordance with the manufacturer’s instructions. The lysis buffer contained 25 mM Tris-EDTA, 10% glycerol, 2 mM dithiothreitol, and 1% Triton X-100. All manipulations with cells were carried out on ice or at 4 °C. Each 10 minutes of incubation, cells washed with PBS were harvested from two different wells for analysis. Cells detached by washing and attached cells were combined during the lysis. Luminescence was measured using an Infinite 200 plate reader.

### 2.11. GSH/GSSG Assay

The level of total (GSH + GSSG) and oxidized glutathione (GSSG) in cells was determined as described in [35], except that the lysis buffer contained 1% Triton X-100. Cells detached by washing and attached cells were combined during the lysis. The accumulation of 5-thio-2-nitrobenzoic acid was traced by absorbance at 405 nm using a plate reader.

### 2.12. mBCl Assay

To evaluate an activity of GSH-S-transferase (GST) in the cells [36], a day after being seeded in microplates, cultures were treated with DDC, B_12b_, and their combination. After the incubation, cells were washed two times and stained in HBSS with monochlorbimane (mBCl, 60 μM). The kinetics of fluorescence was measured at ex 360 nm/em 465 nm on a multiplate reader Infinite 200. A decrease in fluorescence after 10-min preincubation of cells with 5 mM ClDNB or 100 μM NEM (1 h) served as a negative control. The signals were normalized to the cell number estimated by the crystal violet assay after fluorescence measurements.

### 2.13. Immunoblotting

A day after plating, cells grown in T-25 flasks (1.5 × 10^6^) were treated with DDC or DDC + B_12b_, incubated for 1–4 h, washed twice from culture medium with ice-cold PBS, and lysed in lysis buffer (50 mM Tris-HCl (pH 7.4), 150 mM NaCl, 1% Triton X-100, 0.1% SDS, 1 mM EDTA, 1 mM Na_3_VO_4_, and 1 mM NaF) supplemented with proteinase/phosphatase inhibitors. The extracts were incubated on ice for 30 min and centrifuged at 13,000× *g* for 5 min at 4 °C. The supernatants were collected and quantified for protein concentration by using the Bradford protein assay. Then, the supernatants were solubilized by 4× Laemmli sample buffer (Bio-Rad, Hercules, CA, USA). To determine the level of proteins in cell lysate, samples were heated to 95 °C for 5 min and applied to the gel. Protein samples were separated by 12.5% SDS–PAGE and transferred to a nitrocellulose membrane at 300 mA for 1 h. The membrane was blocked in a Roti-block solution for 1 h at room temperature and incubated with the primary antibody at 4 °C overnight and then with an HRP-conjugated secondary antibody. The ER Stress antibody Kit and the polyclonal LC3A/B antibody were from Cell Signaling (Danvers, MA, USA). The β-tubulin antibody (1:1000 dilution; Cell Signaling, Danvers, MA, USA) was used as a loading control. The blot was detected by an ECL detection system (ChemiDoc Touch Imaging System, Bio-Rad). Protein bands were quantified by densitometry (Image Lab program).

As a positive control of autophagy, HEp-2 cells were seeded in a Petri dish 146 mm in diameter at a density of 10,000/cm^2^, and twenty hours after the seeding, the serum containing culture medium was removed and replaced by a fresh medium (Gibco DMEM A1443001, Waltham, MA, USA) without serum, glucose, glutamine, and pyruvate (SGGP-starvation) [37], and after 4 h incubation, cells were treated for the analysis as described above.

### 2.14. Statistical Analysis

Each experiment was performed at least three times. All the values represent the means ± s.e.m. The statistical significance of the results was analyzed using the Student’s test for paired experiments. The values of *p* < 0.05 were considered as statistically significant.

## 3. Results

### 3.1. Vacuolization of the Cytoplasm and the Absence of the Signs of Apoptosis and Necrosis Upon the Initiation of Cell Death by the Combination DDC + B_12b_

**Figure 1:**
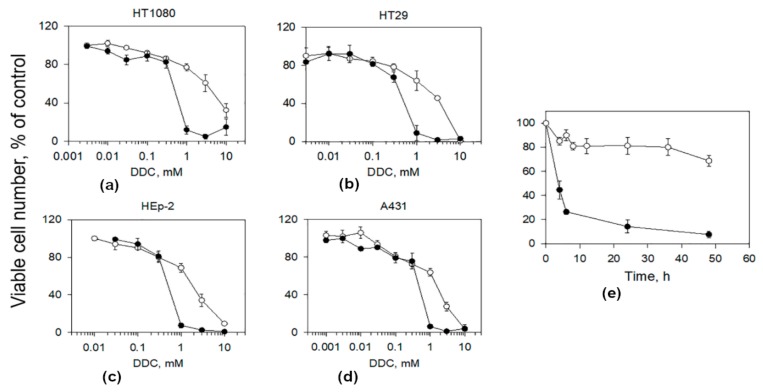
Vitamin B_12b_ enhances the cytotoxic effect of DDC in subconfluent cultures of tumor cells. (**a**–**d**) Enhancement of the cytotoxic effect of DDC by 25 μM B_12b_ toward HT1080, HT29, HEp-2, and A431 cells. (**e**) Dependence of the cytotoxic effect of the combination 1 mM DDC + 25 μM B_12b_ against subconfluent cultures of HEp-2 cells on the exposure time. The components (B_12b_ and DDC) were added simultaneously 24 h after cell seeding. The action of DDC + B_12b_ was interrupted by replacing the culture medium with a fresh growth medium. The cytotoxicity was estimated 48 h after the addition of DDC and B_12b_ (see Materials and methods). Incubation with 1 mM DDC (open circles) and with DDC + B_12b_ (filled circles). The data are the means ± s.e.m. of five separate experiments.

As we have shown earlier, vitamin B_12b_ enhanced the cytotoxic effect of DDC in subconfluent cultures of human A549, A431, HEp-2 cells [20]. In the present work, we found a similar effect in human fibrosarcoma HT1080 and human colon adenocarcinoma HT29 cells (Figure 1a,b). For comparison, Figure 1c,d present the additional data for HEp-2 and A431 cells. DDC used alone at a concentration of 1 mM did not induce cell death and produced a weak cytostatic effect on cell growth. Vitamin B_12b_ was not toxic to these cell lines at concentrations up to 2 mM, and IC_50_ of B_12b_ was 3–3.5 mM. Table 1 gives the IC_50_ values for DDC added alone and in combination with 25 μM B_12b_ on various tumor lines and the Chou-Talalay combination indices (CI) [31]. The CI values for all cell lines studied were considerably less than 1, indicating a strong synergism of the cytotoxic effect of the DDC and B_12b_. The number of dead cells in HT1080 and HT29 cultures increased beginning from 6–8 h after the addition of the combination, just as it happened in A549, A431, HEp-2 cultures [20]. It was found that four to six hours of incubation of cells in a culture medium containing DDC (1 mM) + B_12b_ (25 μM) followed by its replacement with fresh growth medium were sufficient for the initiation of the cytotoxic effect of the combination (Figure 1e). It is seen that the incubation of cells in the presence of DDC alone at a concentration of 1 mM for 48 h did not induce any marked toxic effect. In the following, the mechanism of the cytotoxic effect of the combination DDC + B_12b_ was studied using HEp-2 and A549 cells.

**Figure 2:**
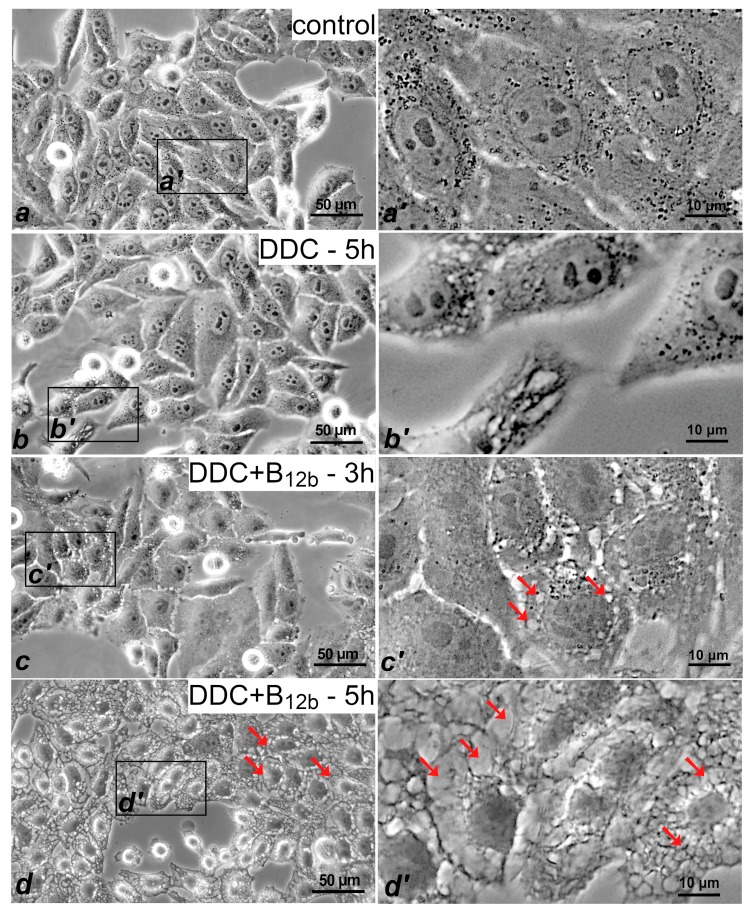
Vacuolization of HEp-2 cells at the stage of death initiation by the combination of 1 mM DDC + 25 μM B_12b_. (**a**) Control (**b**) after 5 h-incubation of cells with 1 mM DDC; (**c**) and (**d**) after 3- and 5-h incubations with DDC + B_12b_. The arrows point to vacuoles in cells treated with DDC + B_12b_. Phase contrast microscopy. (**a’**), (**b’**), (**c’**), and (**d’**):selected areas enlarged in inserts (**a**), (**b**), (**c**), and (**d**).

The death of cells induced by the action of DDC + B_12b_ was preceded by extensive vacuolization of the cytoplasm (Figure 2c,d), which began 2.5–3 h after the addition of the combination. At the initial stages (3 h of incubation), the vacuolization was reversible, with the amount of live cells being the same as in the control (95%), as estimated using the trypan blue assay. By 4–6 h, small and moderate-size vacuoles were transformed into large ones; cell nuclei were squeezed by vacuoles and decreased in size. Cell blebbing and aberrant chromatin distribution, which are the characteristic signs of apoptosis, were not observed. This process of cytoplasm vacuolization realized without the morphological signs of apoptosis was characteristic for all cells studied (Figure S1). DDC (1 mM) alone also induced the appearance of small vacuoles in some cells by 4–6 h of the incubation (Figure 2b); however, after 24 h of the incubation with DDC, vacuolated cells were not observed, and the amount of live cells was as in the control (95%).

**Figure 3:**
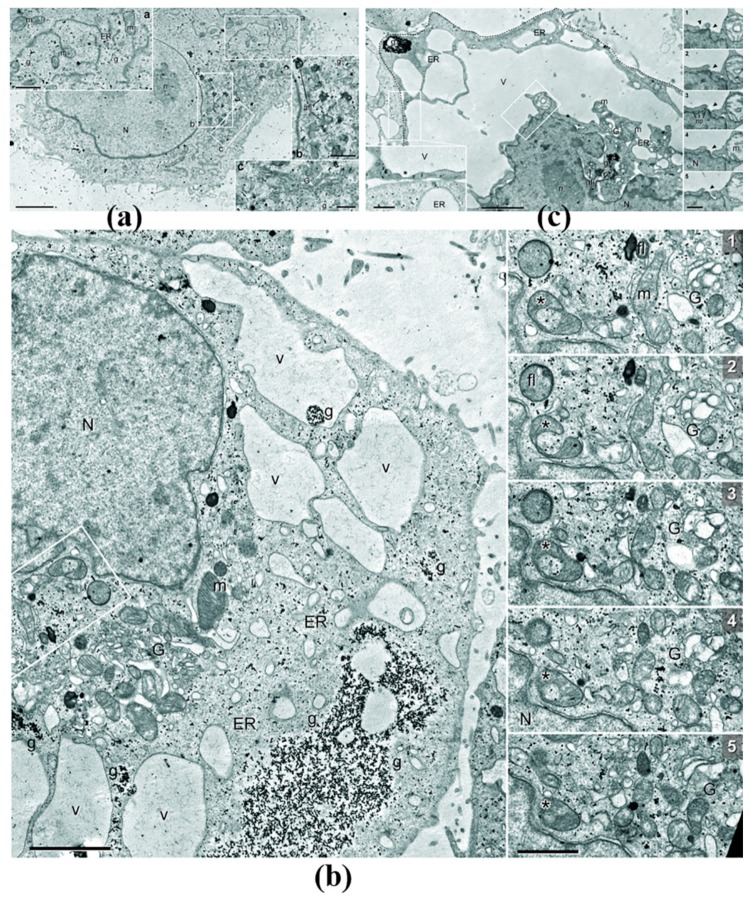
Changes in the ultrastructure of HEp-2 cells caused by incubation for 4 h in the culture medium containing 1 mM DDC and 25 μM B_12b_. (**a**) Ultrastructure of a control cell. Selected areas enlarged in inserts a, b, and c. Scale bars: 3 μm in the overview, 1 μm in a and b, and 0.5 μm in c. (**b**) Ultrastructure of a cell with moderate changes after incubation. Scale bars: 2 μm in the overview and 1 μm in series. (**c**) Ultrastructure of a cell with severe changes after the incubation. For distinguishing the cell from the extracellular space, the plasma membrane of the cell is marked with a dashed line. Scale bars: 2 μm in the overview and 0.5 μm in the left insert and in series. N, nucleus; n, nucleolus; ER, endoplasmic reticulum; G, Golgi apparatus; m, mitochondria; g, glycogen granules; fl, secondary lysosomes; afl, autophagolysosomes; v, vacuole-like ER sacks.

The transmission electron microscope (TEM) examination showed that mitochondria in control cells had distinct intact cristae (Figure 3a), and the endoplasmic reticulum (ER) had a typical structure. By 4–6 h of the incubation with DDC + B_12b_, cells with moderate and severe ultrastructural changes (Figure 3b,c) were seen. In cells with moderate changes, the swelling of cisterns of the ER and Golgi apparatus occurred, flakes and unstructured fibers were seen inside swollen ER cisterns, a net of ER typical of control cells disappeared, and the shape of the cell nucleus changed slightly but without fragmentation typical of apoptosis. The number of autophagosomes or secondary lysosomes did not increase as compared with control cells. Although in some mitochondria, a clearance of the matrix was observed, the structure of a large part of mitochondria did not differ from the control. U-shaped autophagosome-like mitochondria engulfing neighboring cytoplasmic structures were sometimes seen. In cells with severe changes, the deformation of nuclei and a decrease in their volume followed by chromatin condensation occurred; however, the fragmentation of the nuclear material typical of apoptosis was not observed. The increase in the electron density of narrow compartments of the cytoplasm was accompanied by a strong extension of the lumen of ER cisterns. In the interior of ER cisterns, no floccular material was seen. The extension of the ER lumen was accompanied by the fusion of the perinuclear structures with ER cisterns; however, nuclear pores were retained (Figure S2). The Golgi apparatus significantly decreased in size and appeared as small aggregations of densely adjacent cisterns. Glycogen granules tended to agglomerate into dense local aggregations, in contrast to their homogenous distribution in control cells. In cells with giant vacuoles, swelling and clearance of the matrix and disorganization of cristae were observed in mitochondria; in addition, the number of secondary lysosomes decreased. Swollen cisterns of ER at the cell periphery often were in close contact with the plasma membrane, and the distance between the boundary of ER and the extracellular space in these regions was no greater than 10 nm.

On the whole, the electron microscopy examination indicated the lack of the signs of apoptosis and autophagy during the DDC + B_12b_-induced initiation of HEp-2 cell death, minor morphological changes in mitochondria, as well as intensive formation of vacuoles from ER and their transformation into giant vacuoles, which caused disturbances in the nucleus structure and in the distribution of intracellular organelles.

**Figure 4:**
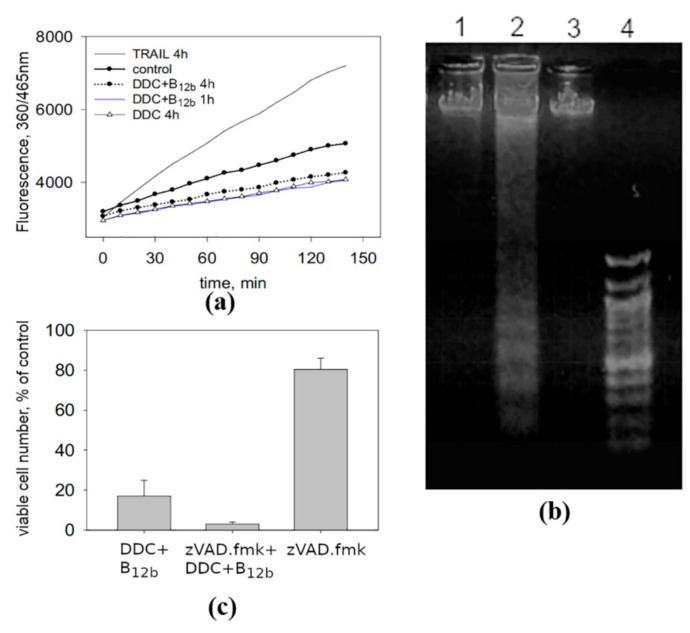
The absence of signs of apoptosis during initiation of cell death by a combination of 1 mM DDC with 25 μM B_12b_. (**a**) Inhibition of the activity of caspase-3 in HEp-2 cells incubated with DDC + B_12b_. As a positive control, the recombinant protein izTRAIL significantly increased the activity of caspase 3. (**b**) The absence of internucleosomal DNA fragmentation in HEp-2 cells treated by the combination DDC + B_12b_. 1—untreated control cells; 2—cells after 24 h of incubation with a combination of 1 mM dithiothriethol + 25 μM B_12b_, which causes apoptosis [18] (positive control); 3—cells after 24 h of incubation with DDC + B_12b_; 4—molecular weight markers. (**c**) The pan-caspase inhibitor zVAD.fmk did not protect cells from death induced by the combination DDC + B_12b_. zVAD.fmk (50 μM) was added to culture medium 1.5 h before the addition of DDC + B_12b_ for 6 h, after which the medium was replaced with fresh one and then after 48-h cultivation the cytotoxicity was evaluated. The data are the means ± s.e.m. of three separate experiments.

That the type of cell death induced by the DDC + B_12b_ combination differs from apoptosis is confirmed by the suppression of the activity of caspase 3 in cells incubated with the combination, the absence of DNA internucleosomal fragmentation (Figure 4a,b), and the lack of the protective effect of the pan-caspase inhibitor zVAD.fmk (50 µM) after its addition into the medium together with DDC and B_12b_ (Figure 4c). Evidence indicating the absence of the signs of apoptotic death (caspase-3 inhibition, lack of membrane blebbing and nuclear fragmentation, and no protective effect of zVAD.fmk) during the DDC + B_12b_-induced initiation of the cytotoxic effect was also observed in A549 cells (Figure S3).

### 3.2. Absence of the Signs of Autophagy During Cell Vacuolization Induced by DDC + B_12b_

**Figure 5:**
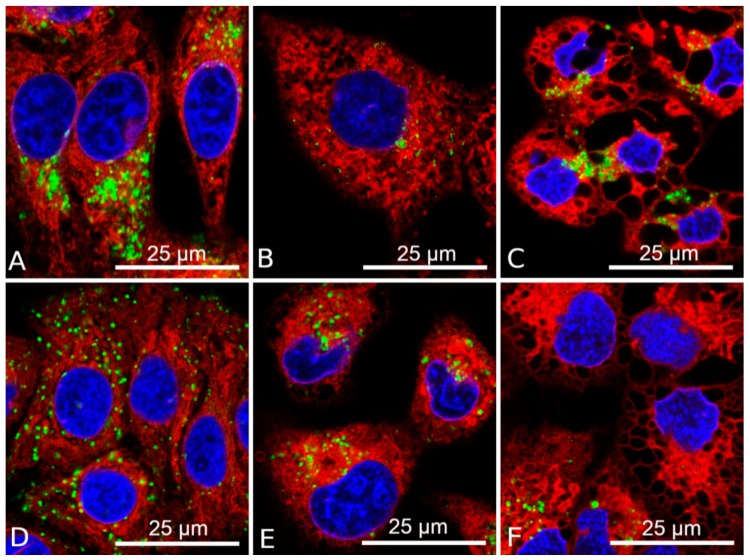
Confocal microscopy images of ER-mediated vacuolization of НЕр-2 (**a**–**c**) and A549 (**d**–**f**) cells after 6 h of incubation with 1 mM DDC + 25 µM B_12b_. (**a**), (**d**) control cells; (**b**), (**e**) cells incubated for 6 h with 1 mM DDC; (**c**), (**f**) cells incubated for 6 h with DDC + B_12b_. Staining with H342 (1 µg/mL), ER-Tracker Red (1 µM), and LysoTracker Green (0.2 µM).

**Figure 6:**
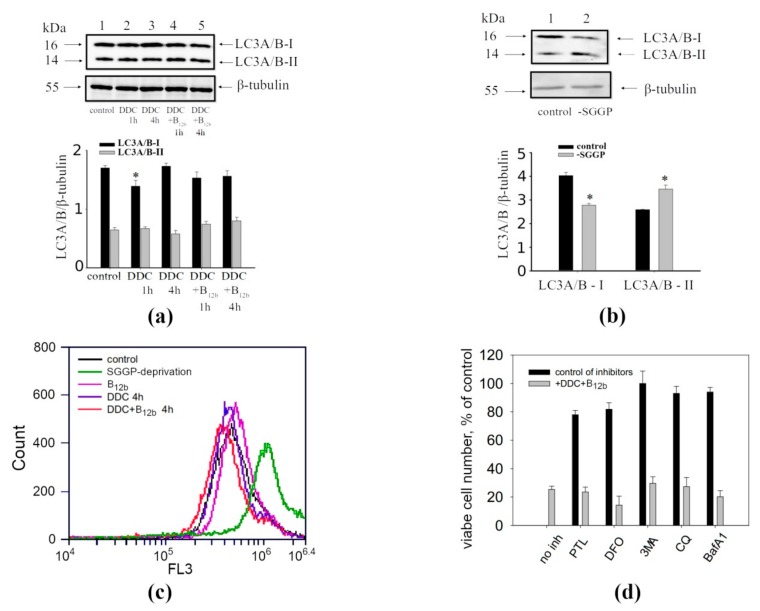
Absence of the signs of autophagy and the involvement of lysosomes in the induction of death of HEp-2 cells by the combination 1 mM DDC + 25 µM B_12b_. (**a**) Immunoblot analysis of autophagy markers in HEp-2 cells after the 1-h and 4-h incubation with DDC (1 mM) or DDC + B_12b_; (**b**) immunoblot analysis of autophagy markers in HEp-2 cells after the 4-h incubation in SGGP-deprived medium; (**c**) FL3 fluorescence of HEp-2 cells loaded with AO in untreated cultures, after 4-h starvation in SGGP-free medium, after 4-h incubation with DDC, B_12b_, and DDC + B_12b_; (**d**) The chelators of intracellular iron ions and autophagy inhibitors did not protect cells during the 6-h incubation with DDC + B_12b_. Cells were incubated in growth medium for 90 min with inhibitors or chelators, then DDC + B_12b_ was added, and after 6-h incubation, medium was replaced by a fresh growth medium without additives. The cytotoxic effect was estimated 48 h after the treatment. *, differences between the samples and the control are significant, *p* < 0.05.

The fluorescence microscopy data obtained using the Lysotracker Green molecular probe confirmed the results of TEM, which showed that lysosomes are not colocalized with vacuoles formed by the action of DDC + B_12b_ in HEp-2 and A549 cells (Figure 5). In the presence of both DDC alone and DDC + B_12b_, the expression of the autophagy markers LC3-I and LC3-II does not markedly change (Figure 6a). For comparison and as a positive control, serum, glucose, glutamine, and pyruvate (SGGP) starvation, a known enhancer of autophagy [37], was used, which initiated the appearance of small vacuoles and the cell death. In this control, a western blot analysis revealed a fall in the expression of LC3-I and an increase in the expression of LC3-II (Figure 6b), indicating the formation of autophagic vacuoles. A flow cytometry analysis of cells incubated for 4 h in culture medium containing DDC + B_12b_ and stained with acridine orange (AO) revealed no increase in the intensity of red fluorescence (FL3) relative to control (untreated) cells (Figure 6c), indicating the lack of autophagy [32]. For comparison, the induction of autophagy by SGGP starvation raised red fluorescence. The inhibitors of autophagy 3-MA, chloroquine, and bafilomycin A1 did not prevent the vacuolization of the cytoplasm and cell death induced by the DDC + B_12b_ (Figure 6d; Figure S4). The same results of flow cytometry and inhibitory analysis were obtained for A549 cells (Figure S5a,b). Taken together, the results presented indicate that, during the induction of cell death by the combination DDC + B_12b_, the signs of autophagy are lacking. The maintenance of the fluorescence intensity of AO-stained cells treated with DDC and B_12b_ indicates that the lysosome membrane remains stable during the initiation of cell death by DDC + B_12b_, which enables one to eliminate oncosis and necroptosis from the list of possible pathways of the death [39]. The facts that the iron chelators DFO (1 mM) and PTL (50 µM) did not protect cells from vacuolization and death (Figure 6d) indicate that the cell death is not associated with ROS formation caused by the release into the cytosol of iron deposited in lysosomes [40]. These data, too, enable one to eliminate ferroptosis from the list of possible mechanisms of cell death induced by DDC + B_12b_.

### 3.3. Maintenance of the Functional State of Mitochondria During the Initiation of Cell Death by DDC + B_12b_

**Figure 7:**
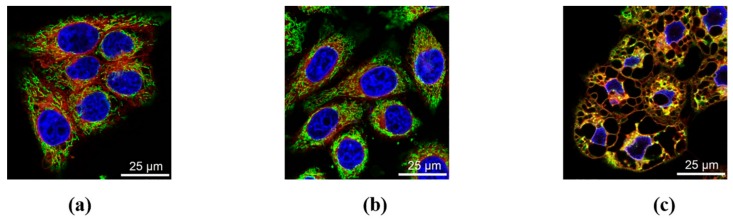
Confocal microscopy images of HEp-2 cells stained with Mitotracker Green (0.2 µM), ER-tracker Red (1 µM), and the H342 (1 μg/mL). (**a**) Control; (**b**) cells after 5-h incubation with 1 mM DDC and (**c**) with 1mM DDC + 25 μM B_12b_. The membranes of vacuoles that appeared in cells were stained with red ER tracker but not with the green Mitotracker.

**Figure 8:**
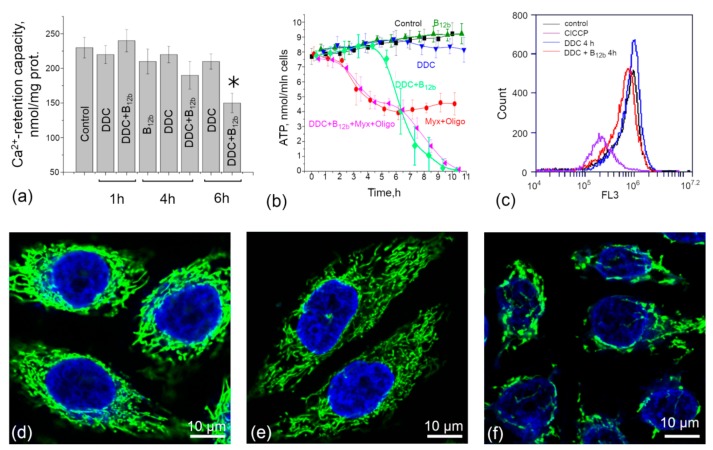
Effect of 1 mM DDC and 25 µM B_12b_ alone and of their combination on the indicators of mitochondrial activity: (**a**) capability of mitochondria to accumulate Ca^2+^ in permeabilized cells; (**b**) the amount of intracellular ATP; (**c**) membrane mitochondrial potential evaluated by flow cytometry of cells loaded with 10 nM DiOC6(3); (**d**–**f**) confocal microscopy images of HEp-2 cells loaded with H342 (1 μg/mL) and 10 nM DiOC6(3); (**d**) control (untreated) cells; (**e**) cells after 4-h incubation with DDC; and (**f**) cells after 4-h incubation with DDC + B_12b_. The concentrations of myxothiazol (Myx) and oligomycin (Oligo) were 20 and 10 µM, respectively. *, differences between the sample and the control are significant, *p* < 0.05.

It is seen from the TEM data that, during the incubation of cells in the presence of DDC + B_12b_, mitochondria almost do not change in size and are not colocalized with vacuoles. The confocal microscopy of cells loaded with the ER tracker Red and Mitotracker Green showed that the membranes of large vacuoles are stained with the ER tracker (Figure 7). By 4–6 h of incubation, a disordering of the mitochondrial network by large vacuoles and the aggregation of mitochondria into dense structures were observed, which at some places were located near vacuoles. We estimated how these changes affect the functional characteristics of mitochondria. Figure 8 shows the effect of incubation of cells with B_12b_ and DDC, taken alone and in combination, on the capability of mitochondria to accumulate Ca^2+^ (a) and generate ATP (b) in cells permeabilized by digitonin. ATP generated by mitochondria was defined as the portion (~50%) of total intracellular ATP sensitive to the mitochondrial inhibitors myxothiazol and oligomycin. As it follows from the figure, neither B_12b_ nor DDC affected the level of intracellular ATP. The effect of B_12b_ plus DDC became visible only after the ~6-h incubation, i.e., after the initiation of cell death. Similarly, mitochondria retained the Ca^2+^-retention capacity (a) till the sixth hour of incubation with DDC + B_12b_, indicating the intactness of mitochondria. This is also evidenced by the fact that mitochondria are capable of maintaining ΔΨ_m_ over the first four hours of incubation of cells with DDC + B_12b_ (Figure 8c–f). The results obtained indicate that, during the initiation of cell death, the functional state of mitochondria is not disturbed.

### 3.4. Intracellular Redox Balance During the Initiation of Cell Death by DDC + B_12b_

**Figure 9:**
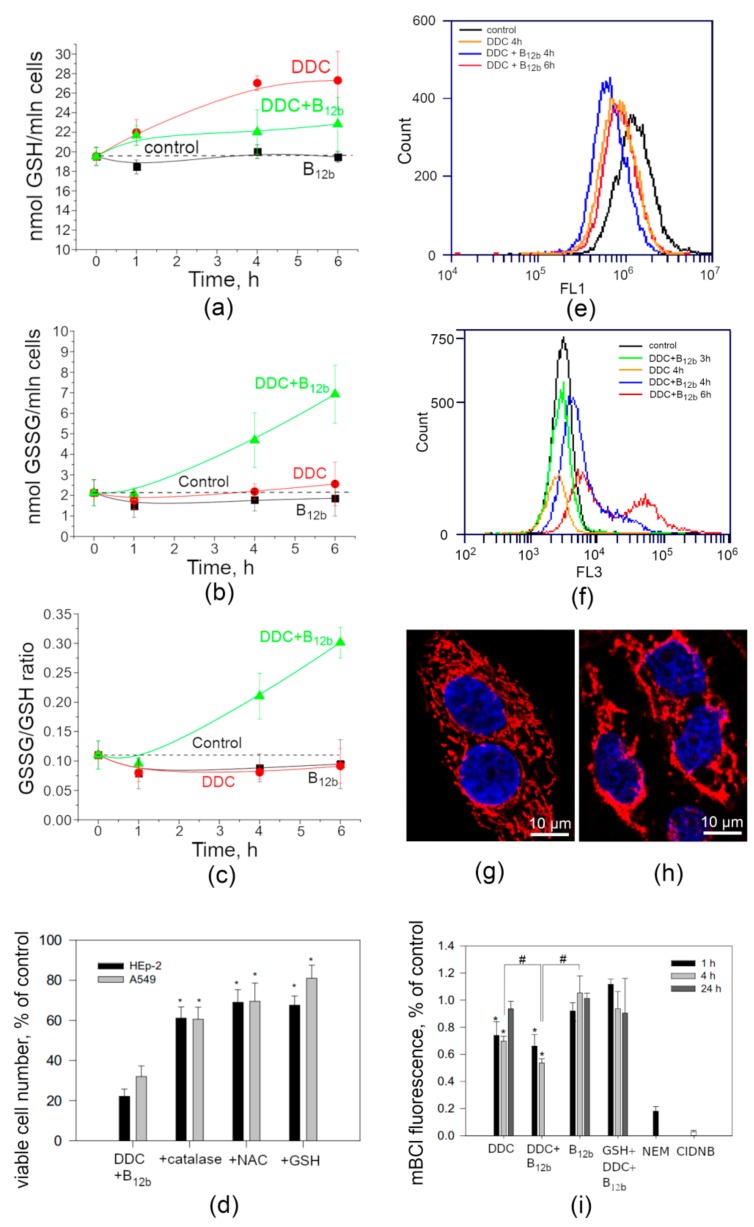
Effect of 1 mM DDC, 25 µM B_12b_, and of their combination on the cell redox balance. (**a**–**c**) Changes in the level of intracellular oxidized and reduced glutathione. At the indicated time, attached and detached HEp-2 cells were harvested and used for the determination of the content of GSH (**a**) and GSSG (**b**), and their ratio (**c**). Each measurement was repeated twice. (**d**) Antioxidants (catalase and thiols) partially inhibit the cytotoxic effect induced by 6-h incubation with DDC + B_12b_. * *p* < 0.05, compared to cells treated with DDC + B_12b_. (**e**), (**f**) Flow cytometry data on oxidative activity in cells loaded with 20 µM DCHFDA (**e**) and 1 µM MitoSOX (**f**). (**g**) and (**h**) Confocal microscopy images of HEp-2 cells stained with H342 and MitoSOX before incubation (**g**) and after 4-h incubation (**h**) with DDC+B_12b_. (**i**) Analysis of mBCl fluorescence in cells incubated with DDC and DDC + B_12b_. After incubation, cells were washed twice and stained with 60 μM mBCl. Cells incubated with 5 mM ClDNB (10 min) served as a negative control. *, differences between samples and the control are significant, *p* < 0.05; #, differences between samples are significant, *p* < 0.05.

Within the first six hours of incubation of cells with DDC + B_12b_, a minor (10%) increase in the level of GSH and a significant (threefold) increase in the level of GSSG and the GSSG/GSH ratio in cells occur. DDC applied alone increased the concentration of GSH by 30–35% and did not affect the level of GSSG and the GSSH/GSH ratio (Figure 9a–c). B_12b_ affected neither GSH nor GSSG. Despite the fact that the level of intracellular GSH did not fall (Figure 9a), the addition of 10 mM exogenous GSH as well as catalase (200 E/mL) and NAC (10 mM) resulted in a much more prominent inhibition of the cytotoxic effect (Figure 9d) and the formation of vacuoles during the 6-h incubation of HEp-2 and A549 cells with the DDC + B_12b_ combination.

It was also found by flow cytometry that the fluorescence of DCHFDA-loaded cells in the first six hours of incubation with DDC and DDC + B_12b_ is not only not enhanced, but is even slightly reduced (Figure 9e). At the same time, when using flow cytometry and confocal microscopy, we observed an increase in the fluorescence intensity of MitoSOX-loaded cells beginning from the fourth hour of their incubation with DDC + B_12b_, which may indicate an increase in the production of oxygen superoxide anion in mitochondria after the initiation of cell death (Figure 9f–h).

Along with the maintenance of the intracellular GSH within 6 h of the action of DDC and DDC + B_12b_, even 1 h after the incubation of cells with these reagents, the fluorescence of mBCl decreased indicating a fall in the GSH-S-transferase (GST) activity. The fluorescence of cells loaded with mBCl after 4 h of incubation in the presence of DDC + B_12b_ was significantly lower than with DDC alone. Twenty-four hours after the incubation of cells with DDC, the fluorescence was the same as in the control (Figure 9i). Note that 10 mM GSH, which protects cells against vacuolization and death in a medium with DDC + B_12b_, also prevented the fall in mBCl fluorescence.

### 3.5. Combination DDC+B_12b_ Induces ER Stress During the Initiation of Cell Death

**Figure 10:**
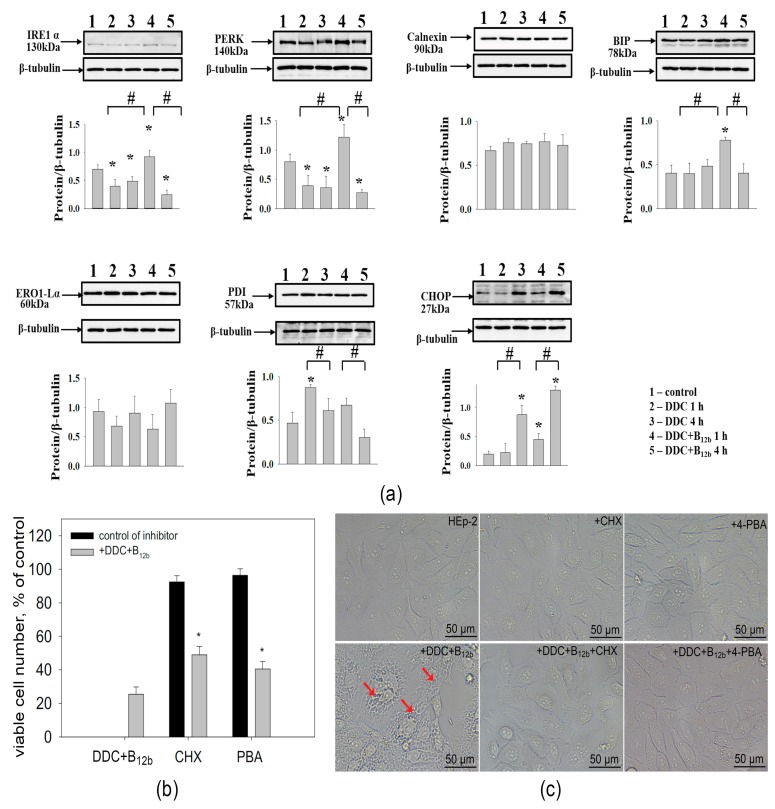
ER stress induced by the action of DDC + B_12b_ causes the disturbance of the synthesis and folding of the protein in HEp-2 cells. Immunoblot analysis of ER-stress markers in HEp-2 cells after 1-h and 4-h incubations with DDC (1 mM) or DDC (1 mM) + B_12b_ (25 µM) (**a**). *, differences are significant compared with control cells; #, significant differences between the groups, *p* < 0.05. The effect of protein synthesis inhibitors on the cytotoxicity of DDC + B_12b_ (**b**) and vacuolization (**c**). Cells were incubated in growth medium for 90 min with 4-PBA or CHX, then DDC + B_12b_ was added, and after 5-h incubation, medium was replaced by a fresh growth medium without additives. The vacuolization was estimated immediately (bright field), and the cytotoxic effect was estimated 48 h after the treatment. *, differences are significant compared to cells incubated without the inhibitors, *p* < 0.05. The arrows point to vacuoles in cells treated with DDC + B_12b_.

The formation of vacuoles from the ER after three to six hours of action of DDC+B_12b_ may be caused by damage to proteins, ER stress, and the unfolded protein response (UPR). The results of the western blot analysis showed that the effect of DDC + B_12b_ at earlier stages is accompanied by a rapid (as early as after 1 h) but transient activation of the ER chaperone BiP (Figure 10). Then, the level of BiP decreased by 4 h to the initial level equal to the control. A similar kinetics was observed with the sensor proteins of the ER stress IRE1 and PERK, which are bound in the inactive state to BiP. The level of expression of another ER stress marker, PDI, did not increase; however, after 1 h of incubation with DDC, it significantly increased. No significant differences in the levels of calnexin and ERO1-α were revealed. The expression of CHOP significantly increased in cells incubated with DDC+B_12b_, particularly by 4 h, where the increase compared with the control was sevenfold; in cells incubated with DDC alone, the expression of CHOP increased fourfold compared with the control. For comparison, the classical ER stress inductor tunicamycin caused a multiple increase in the expression of BiP, IRE1, calnexin, and CHOP, and decrease in PERK in HEp-2 cells by 24 h of incubation (Figure S6). In this case, there were few vacuoles in cells, cell death was not observed later, and the number of live cells after 48-h incubation was about 50% of the control (the cytostatic effect). Incubation of cells with the protein synthesis inhibitor cycloheximide (CHX, 20 µM) decreased cytoplasm vacuolization and cytotoxicity of 6-h incubation with DDC + B_12b_ (Figure 10b,c). The application of the ER stress inhibitor 4-PBA (2 mM), which affects the protein folding and traffic [41], during the initiation of cell death by DDC + B_12b_ also slowed down vacuolization and slightly inhibited the cytotoxic effect. These facts indicate that UPR plays an important role in the initiation of cell death by the combination DDC + B_12b_.

### 3.6. Increase in the Concentration of Intracellular Calcium During the Initiation of Cell Death by DDC + B_12b_

**Figure 11:**
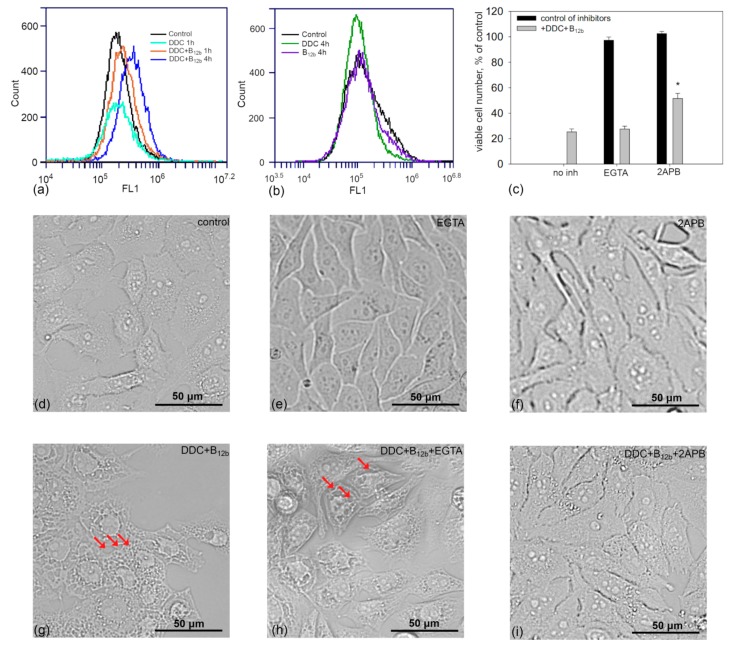
Increase in the concentration of intracellular Ca^2+^ during the initiation of cell death by the combination of 1 mM DDC + 25 μM B_12b_ and the effect of the inhibitor of IP3 receptor and chelator of extracellular calcium on the cytotoxicity of the combination. (**a**) and (**b**) FL1 fluorescence of cells stained with 5 μM Fluo-4AM after incubation with DDC and B_12b_. (**c**) Effect of the IP3 receptor inhibitor and EGTA on the initiation of cell death by the combination DDC+B_12b_ and cell vacuolization (**d**–**i**). Cells were incubated in growth medium for 90 min with 20 μM 2-APB or with 2.5 mM EGTA, then DDC + B_12b_ was added, and after 6-h incubation, medium was replaced by a fresh growth medium without additives. Cytotoxicity was estimated 48 h after the treatment as compared with the control. *, *p* < 0.05, compared to cells treated with DDC + B_12b_. The vacuolization of HEp-2 cells was estimated 6 h after the addition of DDC+B_12b_ (bright field). Control (**d**), 2.5 mM EGTA (**e**), 20 μM 2-APB (**f**); DDC + B_12b_ (**g**), EGTA + DDC + B_12b_ (**h**), and 2-APB + DDC + B_12b_ (**i**). The arrows indicate vacuoles.

It is known that one of the factors inducing the ER stress is the disturbance of the homeostasis of intracellular calcium. Using the flow cytometry method, we found that even after 1 h of incubation of cells with DDC + B_12b_, the intensity of the fluorescence of Fluo-4AM-loaded cells slightly rose (Figure 11). By 4–6 h, the intracellular Ca^2+^ concentration estimated by Fluo-4AM increased two to three times compared with the control. DDC used alone affected the level of intracellular Ca^2+^ to a much lesser extent, whereas the fluorescence of cells after incubation with B_12b_ did not increase. The level of intracellular Ca^2+^ is known to depend, in particular, on the activity of systems of Ca^2+^ release from ER (ryanodine and IP3 receptors). The inhibitor of the IP3 receptor 2-APB (20 µM) markedly suppressed the vacuolization of cells and increased their survival during 6-h incubation of cells with DDC + B_12b_ (Figure 11c,i). The chelation of extracellular Ca^2+^ with 2.5 mM EGTA during 90-min preincubation followed by 6-h incubation with DDC + B_12b_ did not inhibit the vacuolization and the cytotoxicity of DDC + B_12b_ (Figure 11c,h). In the whole, these results indicate the involvement of intracellular Ca^2+^ in the initiation of cell death by the combination of DDC + B_12b_.

## 4. Discussion

We have shown earlier that vitamin B_12b_ significantly increases the toxicity of DDC and that this increase is due to the formation of sulfones and sulfoxides of DSF from DDC, which is catalyzed by B_12b_ [20]. In the present work, we report extended data on an increased cytotoxic effect of DDC combined with B_12b_ on tumor cell lines in subconfluent cultures. The fact that the Chou-Talalay combination index [31] for all cell lines examined was 0.11–0.24, which is less than 1, indicates a high degree of synergism of the action of DDC and B_12b_ used in combination.

It is known that the cytotoxic effect of thiocarbamates such as DDC and DSF is considerably enhanced in the presence of ions of copper and other metals of transient valence [21,22,23,24,25,26,27]. We showed that the enhancement of the cytotoxicity of DDC combined with B_12b_ did not depend on copper ions, although the effect of DDC alone as well as of DSF was strengthened many times by copper ions [20]. This fact indicates that the mechanism of cytotoxicity of DDC + B_12b_ may be different from the mechanism of cytotoxicity of DDC used alone.

In the present work, we showed that the type of cell death initiated by the combination DDC + B_12b_ differs from apoptosis, autophagy, ferroptosis, and necroptosis. It is known that DTC produces either the antiapoptotic or the proapoptotic action depending on concentration, cell type, and the presence of metal ions [24,25,42]. The authors of most works concerned with the cytotoxic effect of DTC in combination with copper or zinc ions found that cell death occurred by apoptosis [24,25,26,27,42,43,44]; however, the data of other authors indicate that the cell death can occur by pathways other than apoptosis [30,45]. In our experiments, the combination DDC + B_12b_ also induced nonapoptotic cell death. It is known that thiurams cause the modifications and cross-links of SH-groups of cellular proteins, including caspases [42,43,46]. We found that oxidized DSF derivatives produced by DDC + B_12b_ [20] had an inhibitory effect on caspase-3. Taken together, the absence of chromatin fragmentation, the retention of integrity of lysosomes, and the lack of their colocalization with vacuoles originating from ER during the initiation of cell death by the combination DDC + B_12b_, all these features indicate that the induced death is related to the ER stress and proceeds by a paraptosis-like pathway [45].

We found using the staining with mBCl that the fluorescence of cells significantly decreased by 1 and 4 h of the incubation with DDC and DDC + B_12b_. As shown above (Figure 9), the level of intracellular GSH during these incubations was by 30% and 10% more than that in the control cells. Therefore, the decrease in fluorescence of mBCl may indicate a decrease in GST activity even after 1 h of incubation with DDC and DDC + B_12b_. This is consistent with data of other authors who showed that DTC form adducts with GST and inhibit it [43,47]. The GST P-form localized in the ER activates, through glutathionation, ER shaperones, in particular, BiP, PDI, calnexin, calreticulin, endoplasmin, and sarco/endoplasmic reticulum Ca^2+^-ATPase and protects their sulfhydryl groups against oxidation [38]. It may be suggested that, along with the action DDC itself, redox-active DSF oxi-derivatives produced by DDC+B_12b_ entry into cells and induce carbamoylation or/and pro-oxidative damage to some proteins, including GST and ER chaperones. This may lead to the accumulation of defective proteins in ER. An initial increase in the expression of ER stress markers, BiP as well as IRE1 and PERK indicate that one of the mechanisms triggering the ER stress may be the disturbance of protein synthesis and folding. This conclusion is supported by flakes and unstructured fibers seen inside swollen ER cisterns on electron micrographs recorded in our experiments and by the effect of the protein synthesis inhibitor cycloheximide and 4-PBA, a compound favorably affecting the maturation and traffic of proteins [41,48]. The enlargement of vacuoles correlated with the fall in the level of IRE1, PERK, and BiP by 4 h and may indicate that cells are incapable of coping with the consequences of the ER stress. The manifold increase in the level of CHOP in DDC + B_12b_-treated cells by 4 h compared with the control and DDC-treated cells, also points to severe ER stress. A drastic drop in ATP by 6 h of incubation indicates the inability of cells to overcome ER stress induced by the combination, which finally culminated with cell death. It should be emphasized that the enhanced cytotoxic effect of DDC + B_12b_ was caused by the generation of extracellular DSF oxi-derivatives [20], and the suppression of this reaction by GSH and NAC, as well as by catalase, had the most noticeable protective effect on vacuolization, cell survival, and GST activity.

It follows from our results that ER stress is also induced in HEp-2 cells incubated in the presence of DDC alone, which is evidenced by the reversible vacuolization of the cytoplasm and the expression of ER markers. The capacity of DTC to induce ER stress was described in the literature [49,50]; however, there is evidence indicating that DDC is capable of diminishing the ER stress [51], and the DSF metabolite DETC-MeSO blocks the specific pathways of ER stress [52]. As seen from our data, cells in the presence of DDC or the classical ER stress inductor tunicamycin are able to survive despite the increased level of ER stress markers, in particular, BiP, IRE1, calnexin, and CHOP. Southwood et al. (2016) also showed that CHOP is not a prodeath protein, and cells can normally function at high levels of this protein [53]. The lack of massive vacuolization in the presence of DDC correlates with significantly increased PDI expression by 1 h, as opposed to DDC + B_12b_.

The increase in the cytosolic calcium concentration during the initiation of cell death by the combination DDC + B_12b_ may also be one of the reasons for ER stress. As known, DSF affects the activity of sarco/endoplasmic reticulum Ca^2+^-ATPase [54] and can also affect, through the modulation of Ca fluxes, the high-conductance Ca channels involved in the regulation of intracellular calcium concentration [55]. The blocking of Ca exit through IP3R retards these processes, which allows chaperones to cope with UPR at initial stages. The increase in cell survival through the addition of the IP3 receptor inhibitor is in favor of this assumption. The effect of DSF oxi-derivatives on Ca channels and their role in ER stress need further investigation.

We did not reveal the formation of vacuoles from mitochondria, which often occurs in the paraptosis-like cell death [56]. It has been found in some studies that, on induction of paraptosis, mitochondria can retain high functional activity [57]. This feature was also characteristic of cells incubated with DDC + B_12b_; even at the stage of the formation of large vacuoles, we did not observe a decrease in the mitochondrial potential, Ca^2+^ retention capacity, and ATP synthesis. However, after 4 h of incubation, the fluorescence of the MitoSOX probe increased, indicating an enhanced generation of superoxide by mitochondria. Probably, this is associated with their aberrant allocation in the cytoplasm caused by the mechanical pushing apart of organelles by giant vacuoles. The ROS generation in turn can give impetus to the aggravation of consequences of ER stress. However, the application of Tiron, a scavenger of superoxide anion, did not prevent either vacuolization or cell death [20], indicating that this phenomenon plays a secondary role in the chain of intracellular events. During the 4-h incubation with DDC + B_12b_ we observed not an increase but even a fall in the fluorescence of cells loaded with DCHFDA. A possible reason for the fall in fluorescence might be not a decrease in the redox activity in cells, but the inhibition of enzymes participating as cofactors in the oxidation of the 2’,7’-Dichlorodihydrofluorescein, e.g., Cu, Zn-superoxide dismutase [58]. The occurrence of oxidative stress in cells incubated in the presence of DDC + B_12b_ is confirmed by a manifold increase in the level of oxidized glutathione and in the GSSG/GSH ratio. The increase in the activity of GSH, which may be considered as an adaptive response to this stress, might also be one of the reasons for the decrease in DCHFA fluorescence in the cell.

**Figure 12:**
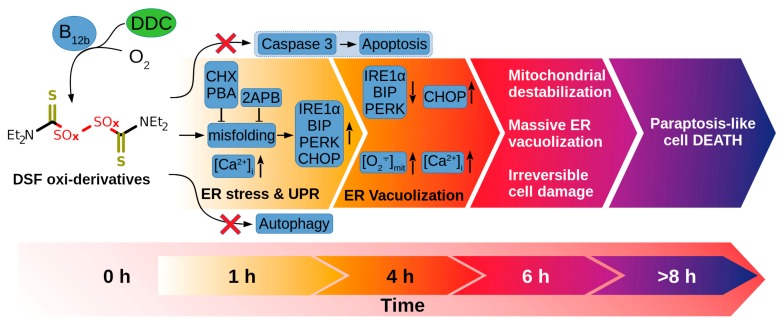
A hypothetic scheme of intracellular events during the initiation of paraptosis-like tumor cell death by the combination DDC + B_12b_ (for details, see the text).

Based on the results obtained, the following mechanism of the cytotoxic action of DDC + B_12b_ can be proposed (Figure 12). DSF oxi-derivatives, presumably sulfoxides and sulfones, cause redox imbalance, an increase in the intracellular calcium concentration, and damage to thiol- and sulfur-containing proteins, including caspase 3, GST, and the enzymes responsible for the activation of autophagy. The accumulation of misfolded proteins in ER results in a drastic extension of the ER cisterns and a massive vacuolization of the cytoplasm (unresolved ER-stress), mechanical deformation of the cell nucleus by vacuoles, condensation of chromatin, a fall in the ATP level, and the disturbance of the allocation of organelles by giant vacuoles, which leads to the initiation of paraptosis-like cell death.

## 5. Conclusions

The data obtained indicate that DDC and B_12b_ used in combination exert a synergistic toxic effect on tumor cells by causing severe ER stress, extensive ER vacuolization, and inhibition of apoptosis, which ultimately leads to the induction of paraptosis-like cell death.

## Figures and Tables

**Table 1:** IC_50_ values for DDC used alone and in combination with 25 μM B_12b_, and CI for different cell lines

Cell Line	IC_50_ for DDC Alone, mM	IC_50_ for DDC Combined with B_12b_, mM	Chou-Talalay Combination Index
A549	2.47 ± 0.64 *	0.4 ± 0.05 *	0.17
A431	1.86 ± 0.21 *	0.43 ± 0.04 *	0.24
HEp-2	2.22 ± 0.24 *	0.46 ± 0.09 *	0.22
HT1080	4.79 ± 0.94	0.5 ± 0.07	0.11
HT29	2.33 ± 0.42	0.53 ± 0.06	0.24

* The results have been reported earlier [38].

## Supplementary Materials

The following are available online at https://www.mdpi.com/2218-273X/10/1/69/s1, Figure S1: Vacuolization of A431, HT29, and A549 cells at the stage of death initiation by the combination of 1 mM DDC + 25 μM B_12b_; Figure S2: Nuclear pores (np) are retained during ER swelling (vacuolization) induced by incubation with DDC + B_12b_; Figure S3: The absence of signs of apoptosis during initiation of A549 cell death by a combination of 1 mM DDC with 25 μM B_12b_; Figure S4: Autophagy inhibitors did not protect HEp-2 cells from vacuolization during the 6-h incubation with DDC+B_12b_; Figure S5: Absence of the signs of autophagy and the involvement of lysosomes in the induction of death of A549 cells by the combination 1 mM DDC + 25 µM B_12b_; Figure S6: Immunoblot analysis of ER-stress markers in HEp-2 cells after 24-h incubation with 2 μM tunicamycin.
