# Supplementary material for: Vitamin B12b Enhances the Cytotoxicity of Diethyldithiocarbamate in a Synergistic Manner, Inducing the Paraptosis-Like Death of Human Larynx Carcinoma Cells
Source: Biomolecules. 2020 Jan 1;10(1):69. doi: 10.3390/biom10010069 (PMC7023477; doi:10.3390/biom10010069)
Supplement: Supplementary file 1 [file biomolecules-10-00069-s001.pdf]

biomolecules

MDPI

Article

Vitamin B_{12b} Enhances the Cytotoxicity of Diethyldithiocarbamate in a Synergistic Manner, Inducing the Paraptosis-Like Death of Human Larynx Carcinoma Cells

Marina Solovieva [1], Yuri Shatalin [1], Roman Fadeev [1], Olga Krestinina [1], Yulia Baburina [1], Alexey Kruglov [1], Ekaterina Kharechkina [1], Margarita Kobyakova [1], Vadim Rogachevsky [2], Elena Shishkova [2], and Vladimir Akatov [1,*]

[1] Institute of Theoretical and Experimental Biophysics, Russian Academy of Sciences, Pushchino, Moscow region 142290, Russia; m_solovieva@iteb.ru (M.S.); yury.shatalin@yandex.ru (Y.S.); fadeevrs@gmail.com (R.F.); ovkres@mail.ru (O.K.); byul@rambler.ru (Y.B.); krugalex@rambler.ru (A.K.); katya.kypri@gmail.com (E.K.); ritaaaaa49@gmail.com (M.K.)

[2] Institute of Cell Biophysics, Russian Academy of Sciences, Pushchino, Moscow region 142290, Russia; vadim_rogachevsky@synapsis.ru (V.R.); shishkova@neuro.nnov.ru (E.S.)

* Correspondence: akatov.vladimir@gmail.com; Tel.: +7495-6237467; Fax: +74967-330553

Supplementary Materials

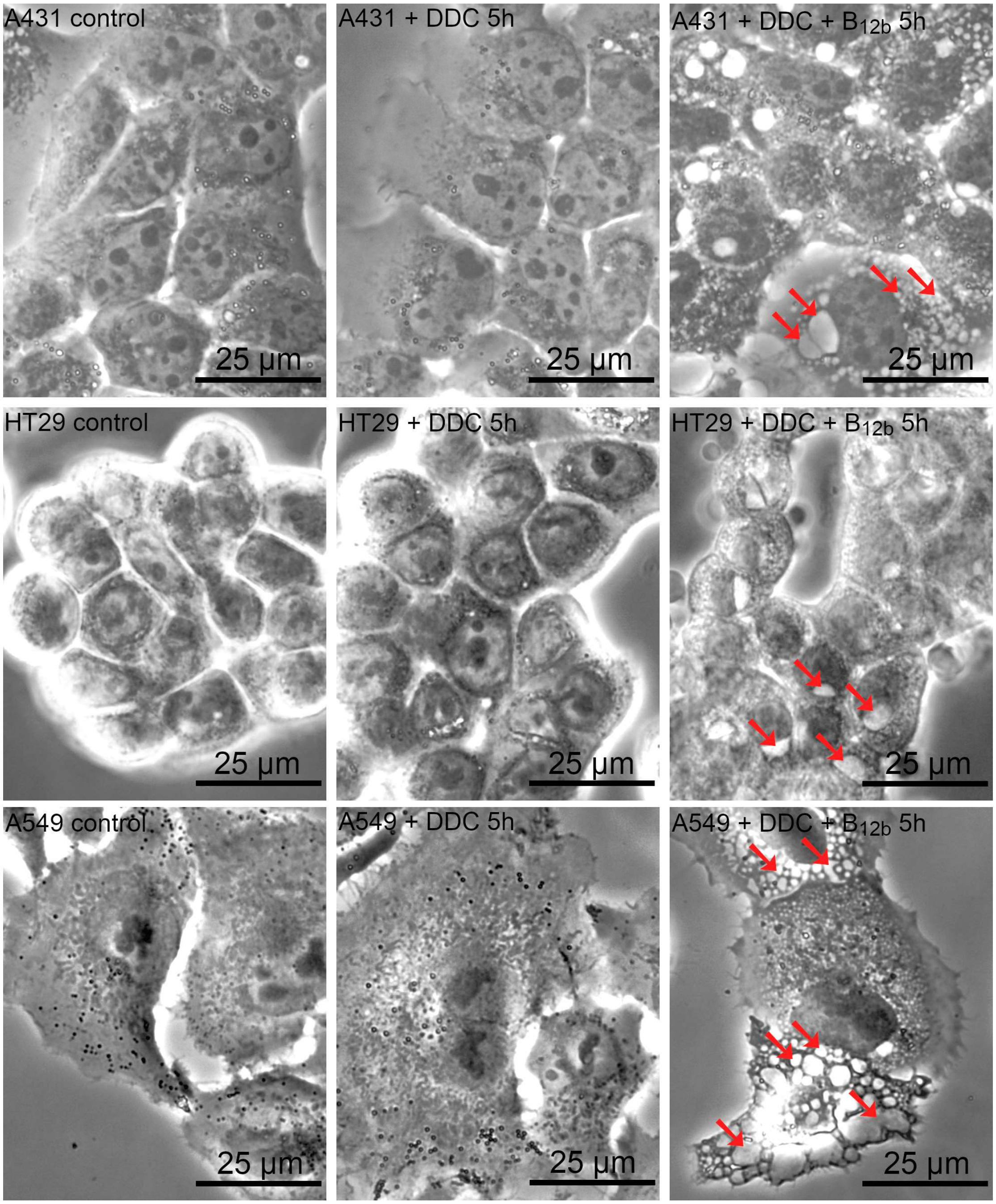

Figure S1. Vacuolization of A431, HT29, and A549 cells at the stage of death initiation by the combination of 1 mM DDC + 25 μM B_{12b}.

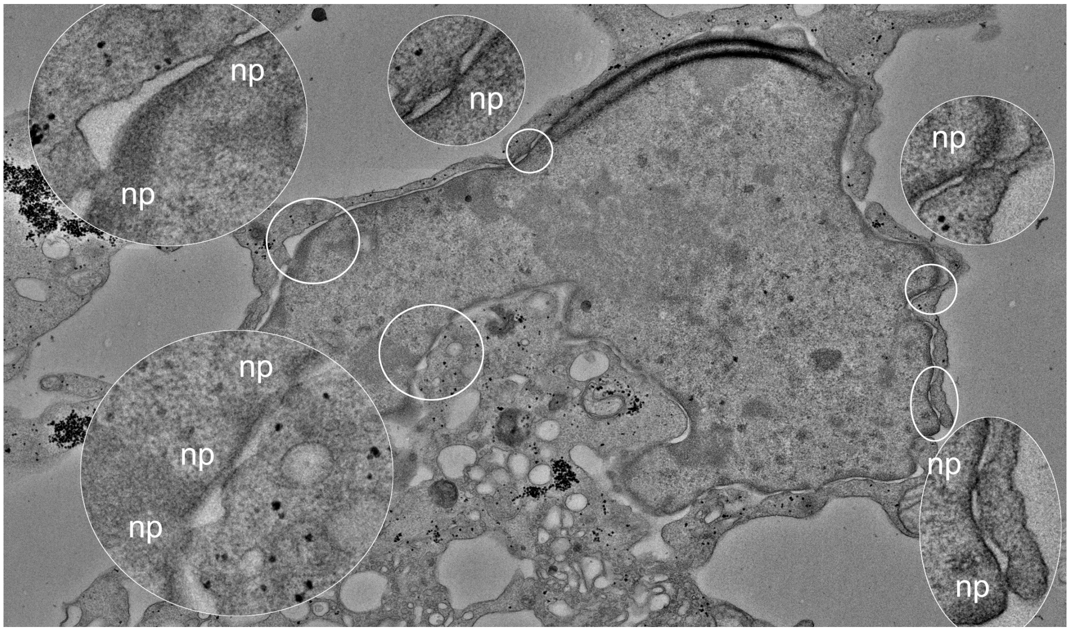

Figure S2. Nuclear pores (np) are retained during ER swelling (vacuolization) induced by incubation with DDC + B_{12b}.

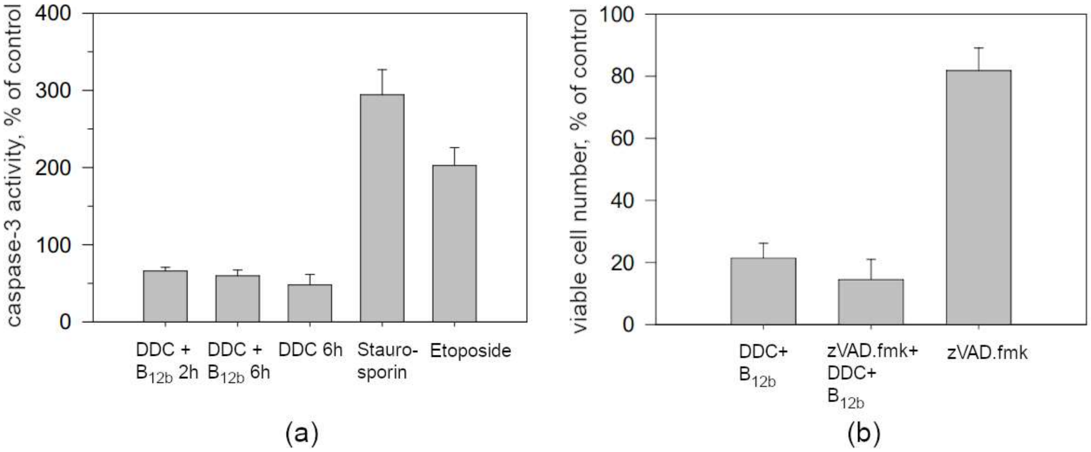

Figure S3. The absence of signs of apoptosis during initiation of A549 cell death by a combination of 1 mM DDC with 25 μM B_{12b}.

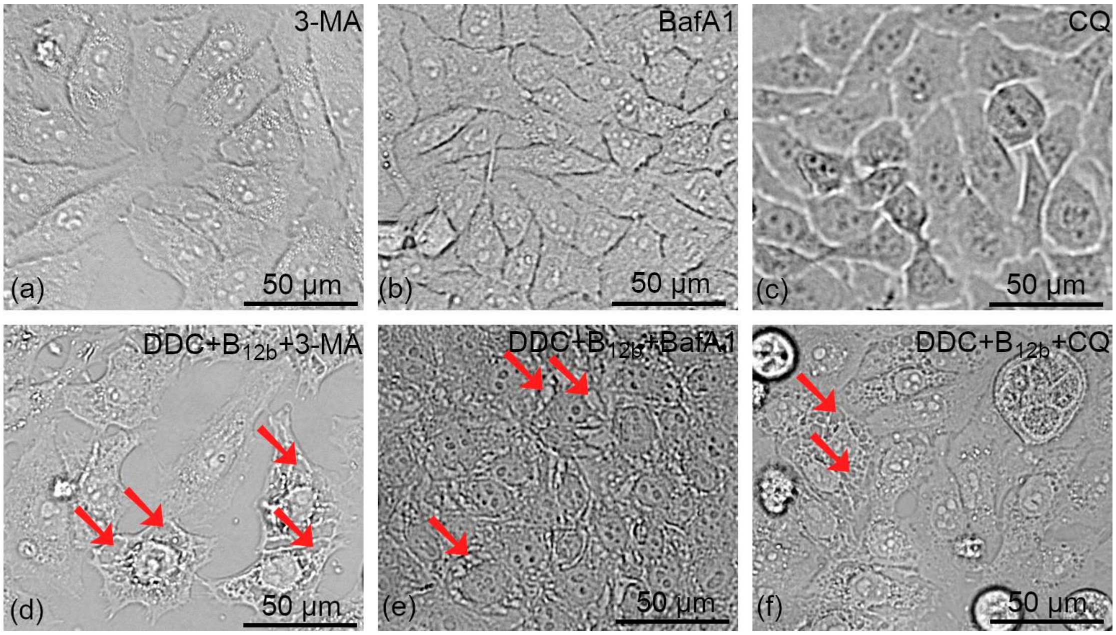

Figure S4. Autophagy inhibitors did not protect HEp-2 cells from vacuolization during the 6-h incubation with DDC+B_{12b}.

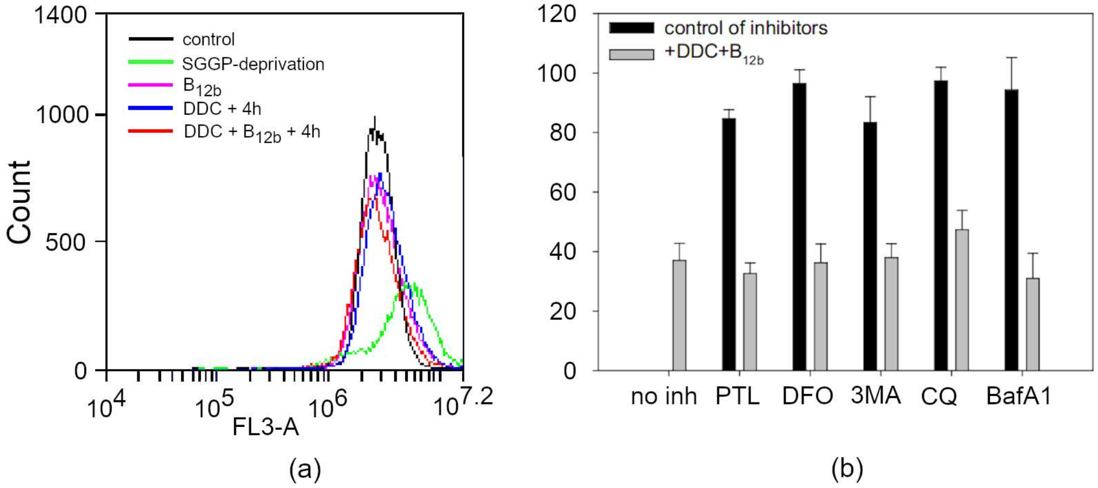

Figure S5. Absence of the signs of autophagy and the involvement of lysosomes in the induction of death of A549 cells by the combination 1 mM DDC + 25 μM B_{12b}.

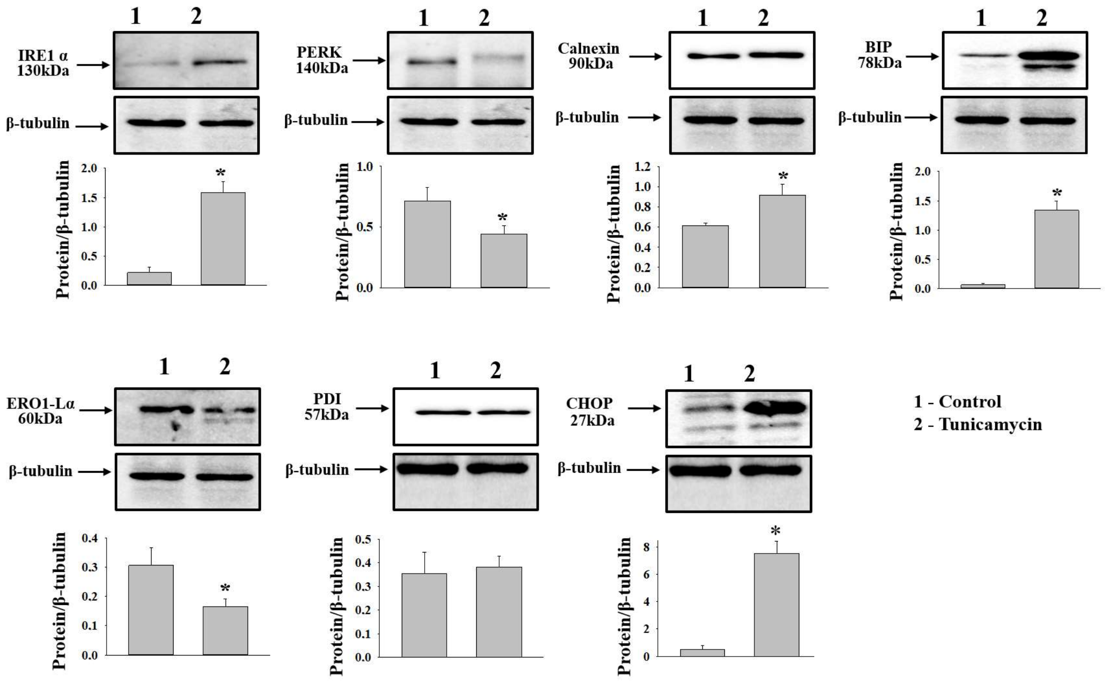

Figure S6. Immunoblot analysis of ER-stress markers in HEp-2 cells after 24-h incubation with 2 μM tunicamycin.
